# My Way to School Through a Camera Lens: Involving Children to Inform a Policy Recommendation on Active School Travel

**DOI:** 10.1177/15248399241255376

**Published:** 2024-06-06

**Authors:** Stina Rutberg, Malin Henriksson, Mathias Andersson, Annie Palstam, Anna-Karin Lindqvist

**Affiliations:** 1 Luleå University of Technology, Luleå, Sweden; 2 The Swedish National Road and Transport Research Institute, Linköping, Sweden; 3 Dalarna University, Falun, Sweden; 4 Sahlgrenska University Hospital, Gothenburg, Sweden; 5 University of Gothenburg, Gothenburg, Sweden

**Keywords:** physical activity/exercise, active school travel, active commuting, active transport, child/adolescent health, photovoice, public health laws/policies, health promotion

## Abstract

Active school travel (AST) is an effective approach for increasing children’s physical activity and independent mobility, but policy supporting AST is lacking. This study aims to explore children’s experiences of AST to inform a policy recommendation. Photovoice methodology with a qualitative approach was applied, with children taking pictures on their way to school. This was followed by focus groups where the children explored their experiences of AST based on their photos. The data were analyzed using qualitative content analysis. The results show that the children valued independent mobility and wanted to be involved in decisions about their travels; they also expressed feelings of increased responsibility and personal growth as a consequence. Although the children recognized areas of improvement regarding infrastructure, especially regarding heavy traffic that jeopardized travel safety, they continued using AST. Finally, the children talked about the value of the health and environmental benefits of AST. Opportunities for friendship, play, and making decisions about their own time were highlighted as important incentives. The benefits from AST are many for children, as well as for society. The result has informed policy recommendations for AST, and the children’s input will be used to communicate the recommendations. Listening to the voices of children could be a steppingstone toward forming future healthy mobility initiatives. In that process, it is key to include children’s perspectives when formulating the AST policy for successful adoption and implementation.

Physical activity contributes to both physical (World Health Organization, 2020) and psychological well-being (Majeed, 2022) and promotes children’s academic achievements (Käll et al., 2014). Globally, as well as in Sweden, children and adolescents are increasingly sedentary. Less than 20% achieve the recommended 60 min of daily physical activity (Guthold et al., 2020). As active school travel (AST) is an effective mean to increase daily routines to physical activity (Larouche et al., 2020; Masoumi et al., 2020), measures to promote it are crucial. In addition, the practice of active travel is often maintained up to adulthood (Kaseva et al., 2023) and can therefore have a lifelong effect on physical activity. Moreover, children’s independent mobility (CIM) is associated with more active travel (Larouche et al., 2020), and CIM is in line with transport policies and the UN Convention on the Rights of the Child. Despite this, CIM has been drastically reduced over the last 50 years (Masoumi et al., 2020). In addition, the distance that children in Sweden are allowed by their parents to cycle has decreased by 40% over the last 30 years (Trafikanalys, 2015). This is partly explained by longer distances to school, due to the free school choice introduced in 1992, and the fact that more parents drive their children to school, making the traffic situation around schools more dangerous (Trafikanalys, 2015). The lack of policies that support AST and CIM constitutes an obstacle to promote AST in Sweden. This is also important as social norms have shown to have an impact on the use of AST and alter the perception of barriers (Forsberg et al., 2020, 2021; Herrador-Colmenero et al., 2017).

## Creating a Policy Recommendation Supporting AST

There is no official policy recommendation regarding AST in Sweden. The National Federation for Traffic Safety, a non-governmental organization, has published guidelines on AST, built on research from the 1960s on children development, stipulating that children must be 12 years old to independently move in traffic environments. Hence, there is a pressing need for recommendations that support children and parents to use AST as several traffic conditions allow children to use AST at any age. Involving children in school travel planning discussions is key to enhancing sustainability (Race et al., 2017). Unfortunately, involvement of children is scarce, and we need to place children at the center of future planning and focus on their needs, rights, and perspectives (Clark et al., 2020). Photovoice is an action-based research method (Catalani & Minkler, 2010) that involves participants as experts both in knowledge development and in dissemination; therefore, it can have a direct impact on policy (Liebenberg, 2022) and have previously been used for informing policy (Pearce et al., 2009). Photovoice studies involving children have been used to address the promotion of children’s physical activity (Liew et al., 2022) and AST in winter climates (Lindqvist et al., 2019). In this study, photovoice is used to include the views of children in an AST recommendation. The aim of this article is to describe (a) children’s experiences and prerequisites for school travel and (b) how the accounts of children informed the recommendation.

## Methods

### Design

To involve children and explore their experiences of school travel, we chose a photovoice methodology with a qualitative approach (Wang & Burris, 1997). The photovoice methodology is recommended as useful input for the formulation or implementation of policy (Pearce et al., 2009).

### Procedure and Participants

Two primary schools in two Swedish municipalities were recruited to participate in the study. We included five different Grade 4 classes in the study. All children in these classes were invited to participate, regardless of transport mode to school. The municipalities differed in terms of geography and demography (see Table 1), which enabled different perspectives on school travel. All children in the selected classes were informed about the study by the researchers (MH and MA). Their parents gave written informed consent for their children to participate. The study was approved by the Swedish Ethical Review Authority (Dnr 2022-01229-01).

**Table 1:** Characteristics of the Municipalities, the School, and the Participating Children

The context	The study was performed in two different municipalities in Sweden, one with approximately 60,000 inhabitants and one with approximately 167,000 inhabitants. In both municipalities, the education level is higher than the national level, and a majority of the inhabitants live in areas with good or very good socioeconomic conditions.
The climate	The climate in these municipalities is characterized by seasonal changes, with colder and darker autumns and winters and warmer and lighter springs and summers. Both municipalities have an average daily temperature in summer of 20–25°C and in winter of −10 to 0°C. One municipality has colder weather with more regular snow and ice during winter months than the other.
The built environment	In Sweden, the condition for active mobility is generally good with well-developed and safe infrastructure, including streetlights. Eight out of 10 children have access to a bicycle. In the bigger municipality, the infrastructure covers 93 miles, and the attitude toward biking is above the national average. In the smaller municipality, cycling paths cover 22 miles.
The primary schools	The schools hosted approximately 500 pupils aged 6–12 years. On average, the pupils lived within 2 kilometers of school, and most of them walked or cycled to school (an average 5- to 10-min bike ride, or 5- to 20-min walk). Pupils who lived more than 4–5 kilometers from school were eligible to ride on the municipal school bus. It was more common in the smaller municipality that the pupils used school bus. Both schools are located in areas with good socioeconomic conditions.
The participating children	One fourth-grade class in one municipality and four fourth-grade classes in the other participated in the study. A total of 43 pupils (14 boys and 29 girls) agreed to participate. Of these, all but one attended the focus group interviews.
The focus groups	Seven focus groups were held with four to eight participants (five mixed-gender groups and two groups of girls). They lasted between 30 and 60 min.

### Data Collection and Data Analysis

The data collection followed the phases described by Wang and Burris (1997). First, the project’s aim and methodology were introduced and discussed with the children. The discussion also included issues of ethics and traffic safety in relation to taking photographs. Next, we asked the children to take pictures that reflected what they perceived as positive, negative, fun, or difficult about their route to school during one week in April.

The photographing was followed by focus groups led by one of the researchers (MH or MA) at the school during school hours with a goal of letting the photos stimulate discussions concerning school travel. The focus group sessions were guided by interview questions, such as “What can you tell me about these pictures?” and “What do these pictures tell us about school travel?” Finally, the groups were asked to pick three pictures that best represented their school trips. In addition to the discussion of the photos, the children were asked to discuss the content and implementation of a policy recommendation for AST.

The data from the focus groups were transcribed and analyzed using qualitative content analysis inspired by Graneheim and Lundman (2004). The focus of the analysis concerning the children’s experiences was their interpretations and discussions of the photos. SR, MH, and A-KL actively participated in the following procedure: (a) The text was first read through several times to obtain a sense of overall data; (b) the text was divided into meaning units; (c) during the abstraction process, the meaning units were coded, and the codes were compared, contrasted, and sorted into preliminary categories, with the authors striving to stay close to the text; (d) the categories were sorted into one main theme and three sub-themes. The final structure was discussed among all authors and presented to the participating children for recognition and approval. Selected quotes and photos are included in the Results section to strengthen the study’s credibility. The next step was for the participants to present the research to both national and local policymakers in the form of photo exhibitions.

## Results

**Figure 1:**
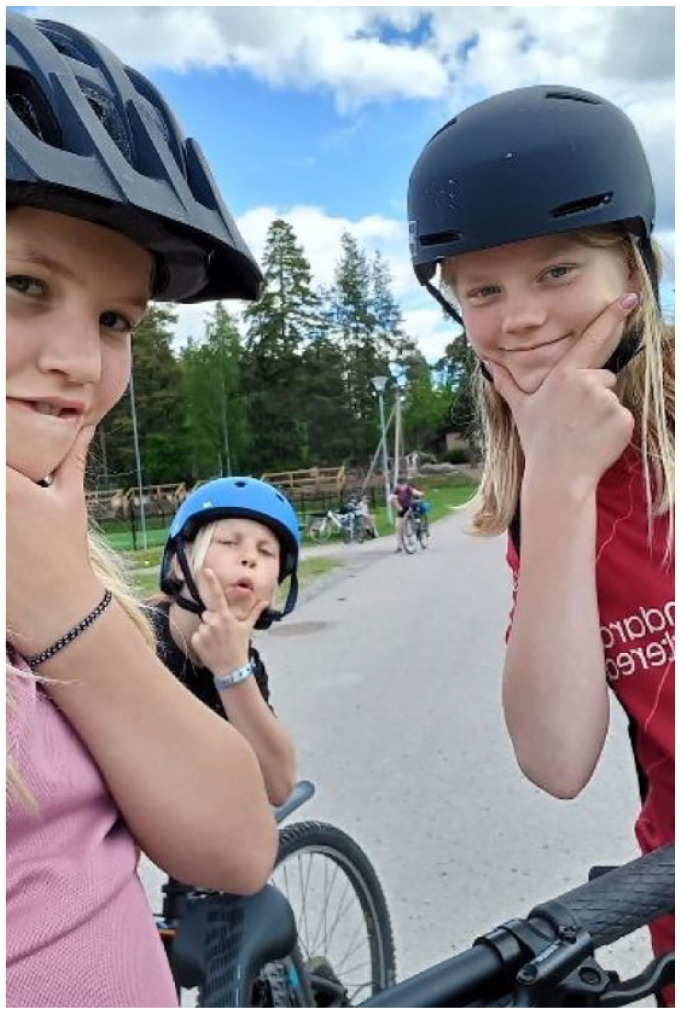
Illustrating the Freedom of Independent Mobility, Including Being Able to Play Football After School

**Figure 2:**
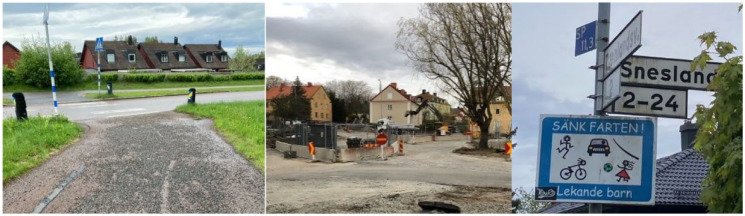
Illustrating Gravel on a Cycle Path and Building Construction as Problems and Signs to Lower Speed as a Suggestion for Improvement

**Figure 3:**
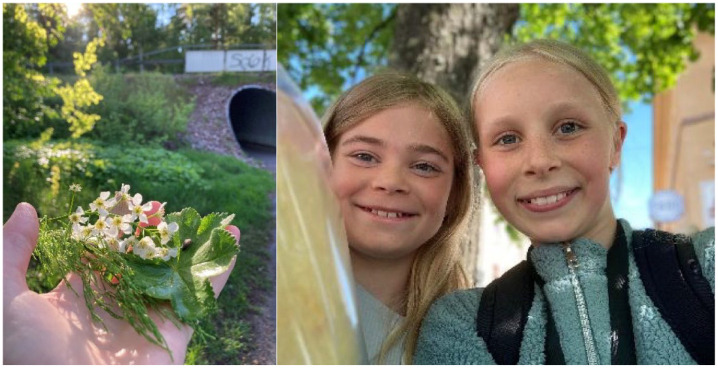
Illustrating the Value of the Environment and the Value of Friendship

The results were formulated into three themes, which in turn provided a basis for the recommendation. Representative photos and quotations from the transcribed text were used, and the quotes were labeled with a pseudonym. The images and stories covered 3 themes: Independent Mobility: Owning the Decision (see Figure 1), Room for improvement - but we do it anyway (see Figure 2), The value for me and the value for the planet (see Figure 3).

### Independent Mobility: Owning the Decision

The children emphasized the importance of being able to travel independently to school and stated a wish to own the decision about their travel mode. Most of the children managed their travel to school by themselves, regardless of travel mode. Being able to go to school independently made them feel responsible and grown up.

Sara: *I remember when I went for the first time all by myself or with a friend or, in any case, without a parent, I felt so cool.*

Jon: *Exactly.*

Klara: *Responsible.*

Sara: *Like it was something really grown up, and you were an adult. (Group 1)*

The children appreciated being able to decide which route to take home and whether to make stops for playing. They also pointed out that being independent made it easier to make after-school plans with friends.

Tilde: *And it’s easier if you want to hang out with a friend or someone else after school if you cycle or walk.*

Olivia: Because when I want to stay on the football field, I usually cycle to school. (Group 7)

During the first years in school, parents or older siblings accompanied the children and taught them how to find their way and manage difficult traffic situations. Most children were allowed to go by themselves when they were 8–9 years old. There were also examples of children who had to travel with their parents, sometimes because the parents found the traffic environment unsafe.

Binta: *I wasn’t allowed to start cycling on my own until a certain age when my mother thought I was good enough. I thought it was boring not being able to ride a bike myself. I wanted to ride a bike myself. (Group 2)*

The children reported that they wanted to make the decision about how to travel to school by themselves but that it often required negotiations between them and their parents. Traffic, distance, and new vs. familiar routes played important parts. They reflected on how it would feel if the school were to make decisions about their school commute and thought that school dictates would be surrounded by a lot of rules, like going straight to school or not being able to choose whom to go with.

### Room for Improvements—But We Do It Anyway

The children stated that even though there was room for improvement in the infrastructure, especially regarding traffic safety, they commuted to school most often using active travel. They described cars sometimes driving carelessly, which made them more attentive at crossings, especially those without traffic lights. They wished that car drivers and bicyclists would pay more attention to school children and expressed fear of being hit by a car. It could be frightening crossing roads with heavy traffic, they noted. The children highlighted construction sites as problematic as they made the traffic situation difficult to overlook, but they also mentioned streets with curves, trees, or buildings where cars back out.

Fanny: *It was blocked off here, so you kind of must go around the whole building if you’re going to school.*

M: *And how do you think that was?*

Fanny: *It’s pretty bad, and Dad thinks it’s a bit scary to let me go on my own when there are excavators and stuff where you have to ride a bike. (Group 4)*

Poorly maintained roads with potholes or gravel were also mentioned as discouraging, as that made it easier to fall with the bike. Lampposts were mentioned as increasing safety, as dark places could be frightening. The children explained that parents sometimes express their worries about the traffic. Parents driving to the schools were also perceived as a problem.

Bert: *There is a lot of traffic around the school. Parents drive their children by car.*

Bettina: *They think their children are more important than other children; therefore, they go by car. (Group 1)*

The ideal route to school, according to the children, involved low levels of traffic and clearly marked pedestrian crossings, especially at traffic lights. It was considered positive when there were a lot of children in the streets, as drivers then tended to proceed more carefully. Signs instructing drivers to slow down, speed bumps, and narrowing streets by painting white lines on them were considered good ways to limit the speed of cars. The children also highlighted values related to aesthetics and joyful encounters. They appreciated beautiful environments, like green areas and spaces that encourage playing along the route to school. Meeting other people and animals was also discussed as positive. In contrast, they also described environments they interpreted as boring or ugly, like industrial areas or empty and desolate places. Also, the weather affected how the children experienced their commute to school. Rain, snow, or high winds made it more difficult to bike or walk. Some of the children also reported that they were not allowed to bike during winter. The children recognized that if all school children walked or biked to school, the walking and cycle paths would be crowded, and there would be a need for more space for active travelers. If everyone were required to bike or walk, children would need to go to the school closest to home to avoid creating a burden for those who live far away. Also, the spaces for bicycle parking at the schools were described as already overcrowded.

### The Value for Me and the Value for the Planet

The children explained the value of using active travel from both individual and global perspectives. They reflected on active transport providing exercise and fresh air and leaving them more alert when arriving at school. They also stated that walking and biking are good for the climate, recognized that cars are bad for the environment, and indicated that commuters should choose the travel mode that is most environmentally favorable.

Felix: *You must take what is best for the climate, and if two modes are equally good for the climate, then you vote. (Group 4)*

The most-discussed incentives for the children to use active travel were friendship and having control of their own time. Having company on the trip to school was depicted as fun, and some children even chose the mode of transport that was best for socializing. Walking was considered favorable for conversations, while bicycling was seen as fast. Even for children who enjoyed sleeping in, leaving home early to be able to walk with friends was perceived as valuable.

Bert: *It’s fun to have some company.*

M: *What happens when you have company?*

Bettina: *You can have more fun. Doing things together is more fun than doing them alone. (Group 1)*

The children described the journey to school as different from the journey home at the end of the school day. The time pressure was less apparent going home from school, and social relations and playing were highlighted even more. The children discussed how long it took them to walk to school and the different routes they could take. They emphasized that the best route to school was often the most direct and fastest route. To save time, the children used shortcuts that sometimes involved crossing bigger roads with more traffic.

## Policy Recommendations: Forming the Future in Healthy Mobility

Building on the children’s experiences and the photographs, the three identified themes informed an AST recommendation covering the value of independent mobility, the personal and planetary value of active transport, and suggestions for improvements, especially regarding traffic safety. The children expressed a desire to make their own decisions about travel modes and highlighted friendship and time as two of the most important incentives for using active travel. By involving children in this study and collecting children’s voices from previous research, as well as conducting a child-impact assessment, both as a process tool and as a final assessment of the policy recommendation, we ensured the children’s viewpoint on AST was integrated into the policy-formulation process. An expert board, including the research group, drafted the initial policy recommendation, which was then circulated for feedback to 75 policy actors, including national and local authorities as well as non-governmental organizations involved in active mobility, transport, planning, children, school issues, among others. Based on the feedback received, a reference group consisting of stakeholders from 15 different organizations, authorities, and the public sector was established. The process of developing the recommendation revealed conflicts in goals, such as balancing the public health benefits of active travel with concerns about traffic safety for children. Discussion about the formulation of the recommendation centered on determining what is best for children. The final version of the recommendation, presented in Table 2, represents an agreement reached among the 15 contributing organizations, incorporating the perspectives of children included in this photovoice study.

**Table 2:** The Policy Recommendation for AST

For children and parents/caregivers	For public actors such as schools, road managers, and traffic and community planners
We recommend that children use active travel (e.g., walking or cycling) all year round, all or part of the way to and from school, starting with preschool, for environmental and health reasons. This should be done in a safe way with an adult for the purpose of learning about the traffic environment but can eventually take place in the company of other children or independently, based on the child and parent together assessing the child’s abilities in relation to the relevant traffic environment.	We recommend that schools encourage students to travel actively and safely to and from school. We recommend that children’s needs and the conditions for their active and safe school travel are considered in: • community planning and the need for accessibility in walking and cycling plans, infrastructure planning for walking and cycling networks and in the location of schools and other important destination points for children • decisions on speed limits in and outside urban areas • decisions on the design of infrastructure where children travel actively • decisions on quality levels and prioritization of maintenance of all infrastructure where children travel actively • decisions to provide opportunities for attractive bicycle parking and reasonable car parking so that those arriving on foot or bicycle can do so in a traffic-safe manner.

## Discussion

To support AST, we must understand how children experience mobility. Their voices present a vital knowledge base. The results of this photovoice study show that the route to school holds many values for children. Listening to their voices could be a steppingstone toward creating future healthy mobility. The analysis shows that children value independent mobility and want to be involved in decisions about travel modes to school, in discussions with their parents. They had many suggestions for infrastructure improvement, especially regarding traffic safety, which they wanted to convey to officials in the municipalities. Despite these issues, they used active travel to a large extent. The children valued both the health and the environmental benefits of active travel and highlighted opportunities for friendship and for making decisions about how to spend their own time as the most important incentives for using active travel. The three sub-themes of this study had direct impact on the national recommendation for AST. The sub-theme “Independent mobility—owning the decision” is visible in the recommendation for children and parents in the formulation that AST can eventually take place independently, based on a joint decision between the child and the parent. “The value for me and the planet” is visible in that AST should be done for environmental and health reasons. The sub-theme “Room for improvement—but we do it anyway” is visible in the recommendation for public actors, focusing on community planning, infrastructure, maintenance, and more attractive bicycle parking spaces.

The children valued independent mobility, and owning the decision on how to travel to school, at what time, and with whom seemed to strengthen their sense of capability and independence. This could be understood in the context of parents being gatekeepers for decisions on AST (Marzi & Reimers, 2018). The promotion of CIM is dependent on the concept of the good parent as someone who lets their children use AST (Forsberg et al., 2020; Silonsaari et al., 2024). When social norms favor healthy mobility in children, parents are more prone to support their children in walking or cycling to school (Forsberg et al., 2021; Saleme & Pang, 2021). Conversely, as described by children in this study, parental perceptions of risk in relation to independent mobility was one barrier restricting walking and cycling. Nevertheless, Herrador-Comenero et al. (2017) have shown that these barriers could be altered by the prevailing social norms in their local community, making social norms crucial for a more comprehensive understanding of how social norms influence health behaviors and independent mobility. Opportunities for making their own decisions about independent travel were not taken for granted by the children in this study, and gaining parents’ trust seemed to be accompanied by personal growth. These findings support previous qualitative findings that children feel capable of making decisions for themselves when traveling independently to school and when considered trustworthy by their parents (Riazi et al., 2021). In all, traveling to school unaccompanied by parents but sometimes in the company of friends is important for nurturing independent and active travelers.

The surrounding environment and the traffic situation on the way to school left room for improvement. Despite this, the children used active travel and were mostly free to travel as they liked, as long as their parents approved the route and travel mode as safe. In line with our findings, the importance of traffic and built environment for perceived traffic safety in AST has been reported previously, with busy roads, in terms of both volume and speed, presented as the main reason for children and their parents to feel unsafe using AST (Amiour et al., 2022). One topic that was raised in the focus groups was the fear of reckless drivers. Of special concern was the traffic situation in the immediate surroundings of the schoolyard, where, the children noted, parents were often the ones driving. It was considered positive when there were a lot of children in the streets, since cars tended to drive more carefully then. This supports previous findings suggesting that a higher number of pedestrians and cyclists render drivers more aware and cautious (Jacobsen, 2015). The topic raised questions as to what would happen if everyone walked or cycled to school. The children reflected on the shortcomings of the present infrastructure, which was not perceived as fully supporting cycling and walking.

In looking at the meanings that children ascribe to their school travels, we found that elements involving individual as well as global benefits stood out. The school commute was regarded as an outlet for playing and socializing with friends beyond the school and home environment. Here, the children especially highlighted the importance of friendship and fun made possible through independent school travel. These findings are in line with previous studies in which children and adolescents described the social benefits of walking and cycling together on their way to and from school (Lindqvist et al., 2019; Silonsaari et al., 2024). In line with previous research, the benefits of being physically active, breathing fresh air, and being close to nature while traveling to school were other values stressed by the children (Rutberg & Lindqvist, 2018; Savolainen et al., 2020). The children in this study also emphasized the environmental benefits of using AST, where climate-friendly behaviors should be the first choice. Previous findings suggest that children who have connections with nature through everyday life experiences also have more environmentally friendly attitudes (Cheng & Monroe, 2012).

### Limitations

The photovoice method is demanding for participants, and there is no guarantee that participants will be empowered and that change will happen simply because we have conducted a study using this approach. Another possible limitation of this study is that the participants might have a higher general interest in concerns regarding AST than other children. It is, however, difficult to speculate on the ways this might have affected the results.

### Implications for Policy

Advocating for a promotional policy over restrictive measures, such as setting age limits for independent bicycling, presents authorities with the opportunity to advocate AST more broadly and foster positive social norms surrounding it. Social norms that endorse healthy mobility can significantly influence parental support for AST. Therefore, policies should strive to cultivate favorable social attitudes toward active travel while also addressing parental concerns about safety.

Policymakers should also acknowledge the manifold social and environmental benefits of AST, which encompass opportunities for building friendships, engaging in physical activity, and climate-friendly behavior. It is imperative that AST recommendations actively promote these advantages as compelling incentives for both children and parents to opt for active travel options.

Furthermore, the findings underscore the need for infrastructure enhancements and traffic safety measures to bolster AST initiatives. Policies must prioritize comprehensive community planning, infrastructure development, and ongoing maintenance, including the implementation of measures aimed at ensuring the safety of walking and cycling routes, particularly for children.

### Implications for Practice and Research

In our study, the application of the photovoice method proved to be successful in capturing children’s voices and perspectives, thereby informing recommendations for AST. Recognizing the crucial role of children in policy adoption, our research underscores the significance of their contribution. Researchers should consider the photovoice method because this process empowers children to (a) articulate the value of independent and AST, (b) propose necessary improvements to increase AST, and (c) participate in formulating recommendations that directly impact their daily lives. Using photovoice in this research area has the potential to ensure the continuation of further knowledge development and improved implementation research to foster policy recommendations.
